# Supplementary material for: A molecular sensor to quantify the localization of proteins, DNA and nanoparticles in cells
Source: Nat Commun. 2020 Sep 8;11:4482. doi: 10.1038/s41467-020-18082-8 (PMC7479595; doi:10.1038/s41467-020-18082-8)
Supplement: Supplementary file 1 — Supplementary Information [file 41467_2020_18082_MOESM1_ESM.pdf]

Supplementary Information

A Molecular Sensor to Quantify the Localization of Proteins, DNA and Nanoparticles in Cells

Laura I FitzGerald[1,2], Luigi Aurelio[1], Moore Chen[1], Daniel Yuen[1], Joshua J Rennick[1,2], Bim Graham[1] & Angus P R Johnston[1,2*]

[1]Monash Institute of Pharmaceutical Sciences, Monash University, Parkville, Victoria, Australia.

[2]ARC Centre of Excellence in Convergent Bio-Nano Science and Technology, Monash University, Parkville, Australia.

Correspondence

Email: angus.johnston@monash.edu

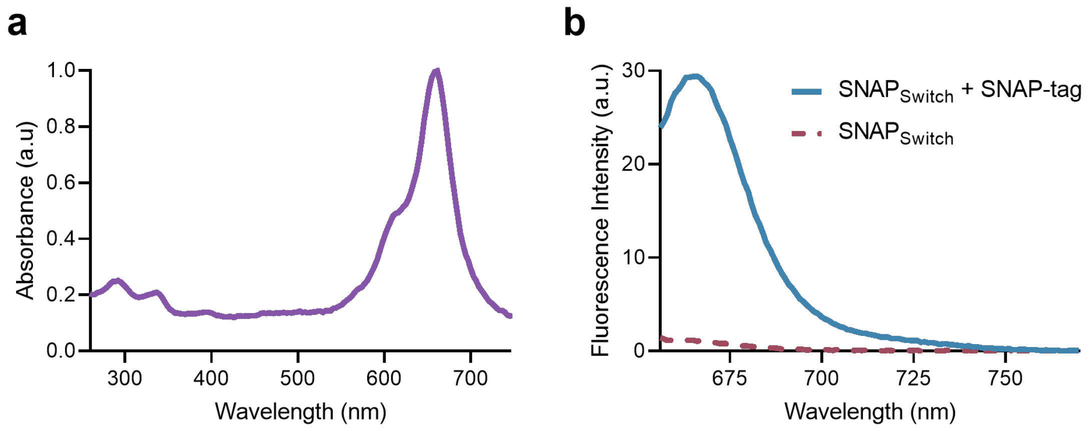

Supplementary Figure 1 – $SNAP_{Switch}$ spectra. (**a**) Normalized absorbance spectra of $SNAP_{Switch}$ in DMSO. The average absorbance of three samples is plotted. (**b**) Fluorescence spectra of 15 μM $SNAP_{Switch}$ in PBS (dotted) or PBS with 75 μM SNAP-tag (solid) treated for 30 minutes at 37°C in PBS. The fluorescence of 1% DMSO in PBS was treated as the background and subtracted from each spectrum. The average of three samples is plotted.

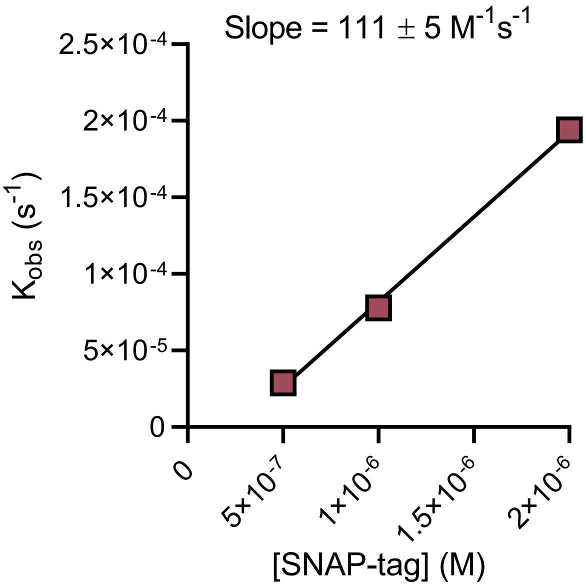

Supplementary Figure 2 – Kinetic analysis of $SNAP_{Switch}$ with SNAP-tag. K_{obs} values obtained from the exponential one-phase association fit from **Figure 2c** plotted versus SNAP-tag concentration.

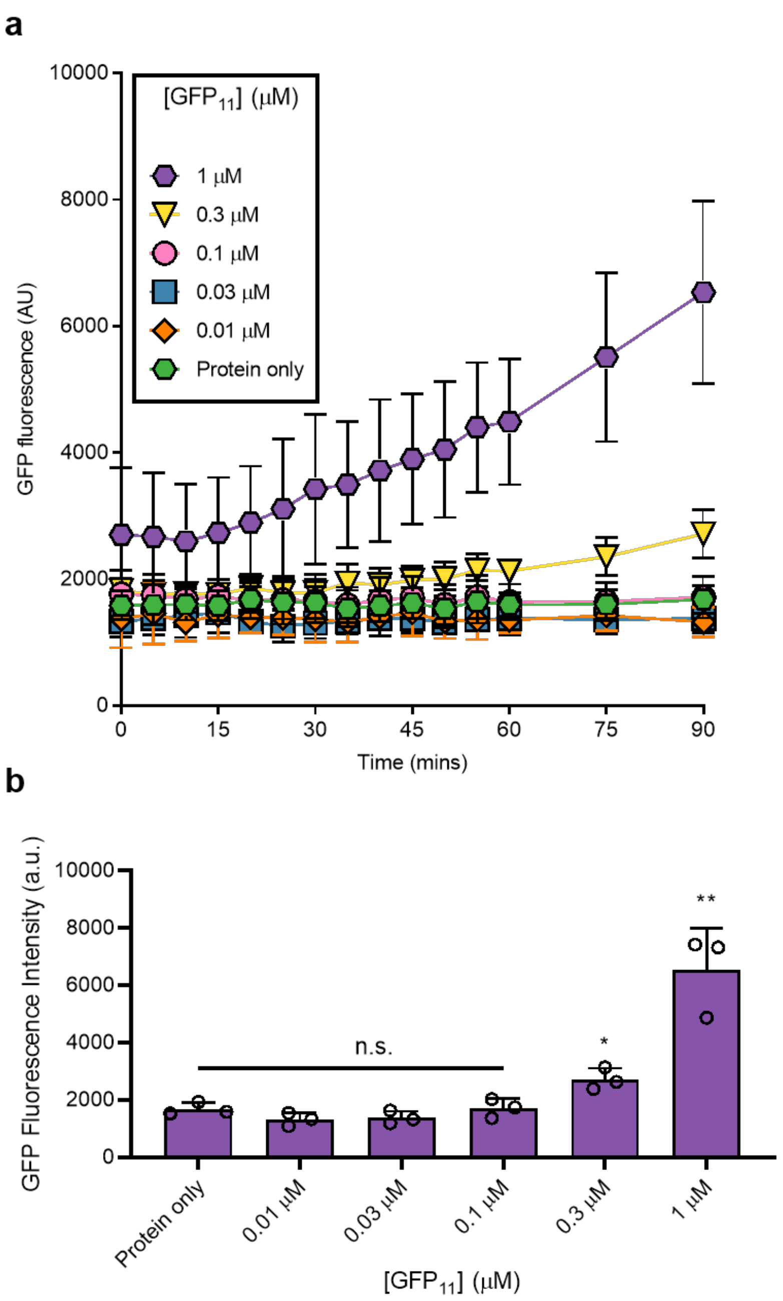

Supplementary Figure 3 – Fluorescence activation in the split-GFP complementation assay in solution. (**a**) 3 μM large fragment (GFP$_{1\text{-}10}$) was incubated with 0 – 3 μM small fragment (GFP$_{11}$) and incubated at 22 °C over 1.5-hours and analysed via a fluorescence plate reader. (**b**) Final fluorescence intensity at 1.5-hours. The average fluorescence intensity of the blank (PBS) was subtracted from each data point. The mean fluorescence is plotted with error bars representing the standard deviation of one experiment in triplicate (n = 3) **(**n.s = non-significant, one-way ANOVA (P = 0.2118). *P = 0.0153, **P = 0.0045, two-tailed, unpaired t-test, n = 3).

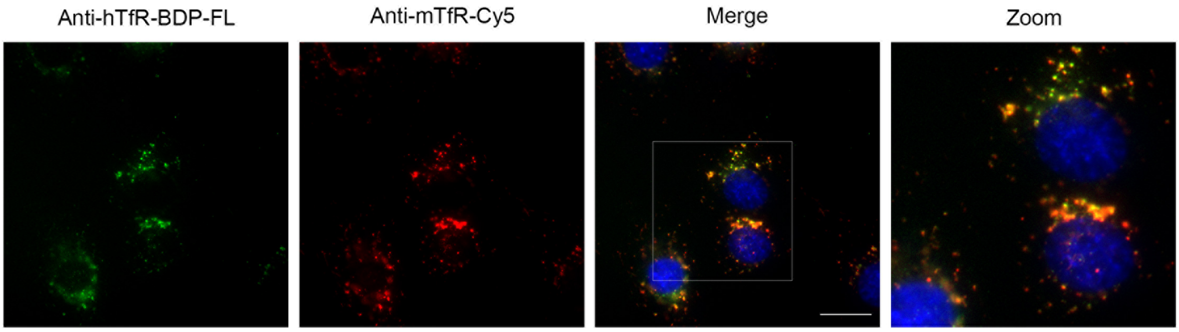

Supplementary Figure 4 – Deconvolved fluorescence microscopy images of 3T3 cells stably expressing human transferrin receptor fused to SNAP-tag (TfR-SNAP) and incubated with antibodies against human (anti-hTfR) and mouse (anti-mTfR) TfR for 1 hour. Pearson correlation coefficient = 0.501, SD = 0.083, n = 3. Scale bar = 20 μm.

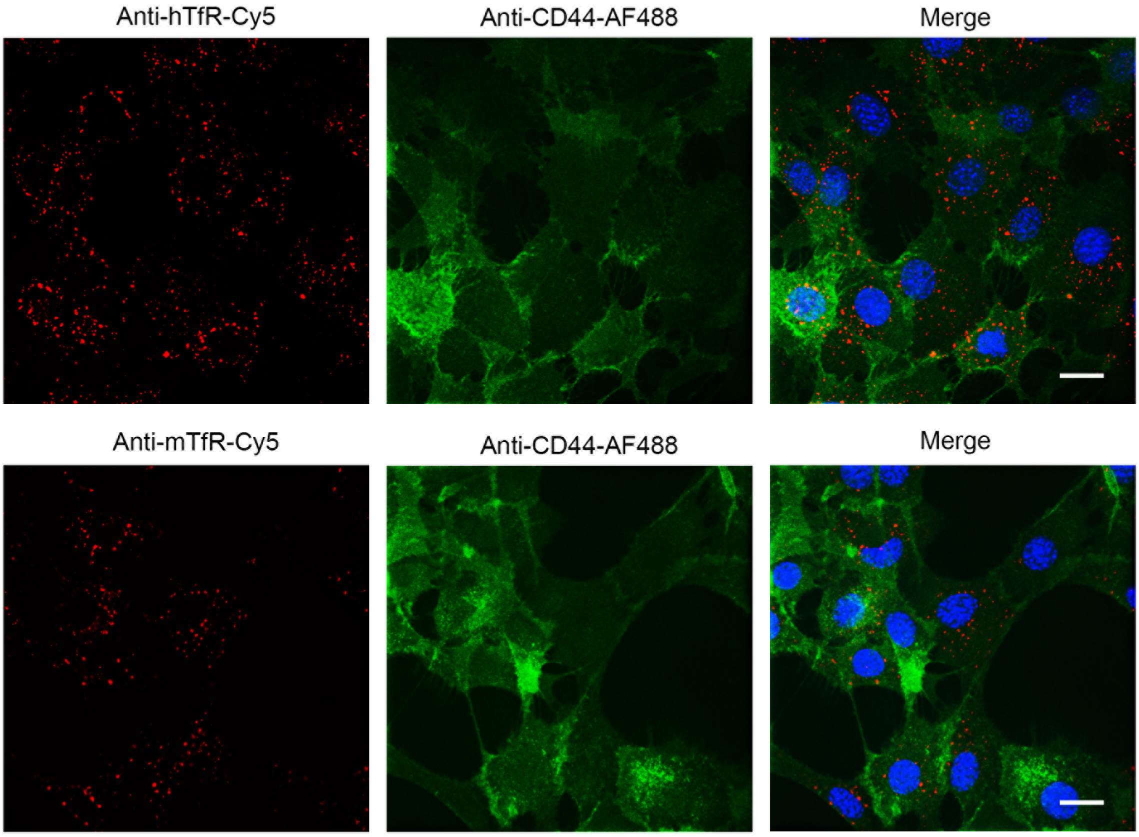

Supplementary Figure 5 – Deconvolved fluorescence microscopy images of 3T3 cells stably expressing human transferrin receptor fused to SNAP-tag (TfR-SNAP) and incubated with antibodies against CD44, human (anti-hTfR) and mouse (anti-mTfR) TfR for 1 hour. The nucleus is stained with Hoechst. Scale bar = 20 μm.

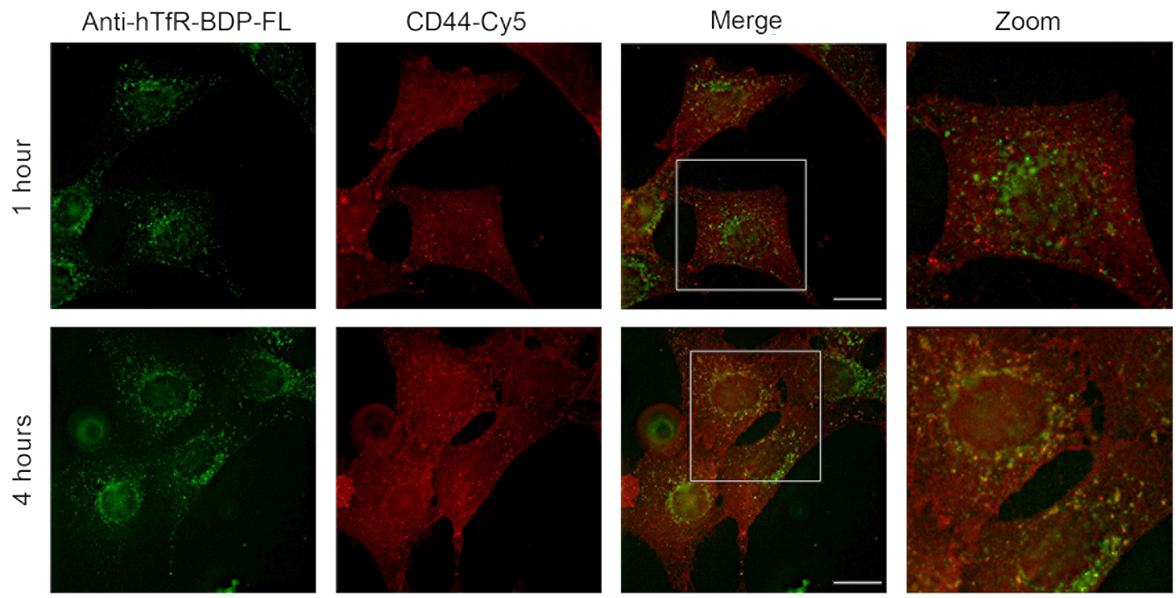

Supplementary Figure 6 – Deconvolved fluorescence microscopy images of 3T3 cells stably expressing human transferrin receptor fused to SNAP-tag (TfR-SNAP) and incubated with antibodies against CD44 and mouse (anti-mTfR) TfR for 1 or 4 hours. Pearson correlation coefficient: 0.238, SD = 0.052 (1 hr) and 0.332, SD = 0.037 (4 hr), n = 3. Scale bar = 20 μm.

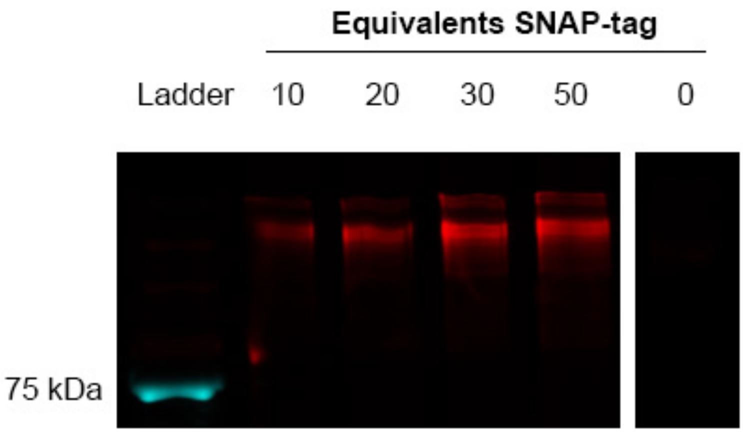

Supplementary Figure 7 – Fluorescence in-gel detection of anti-mouse TfR antibody (anti-mTfR) labelled with $SNAP_{Switch}$ and incubated with 0 – 50 equivalents of SNAP-tag for 1 hour at 37°C.

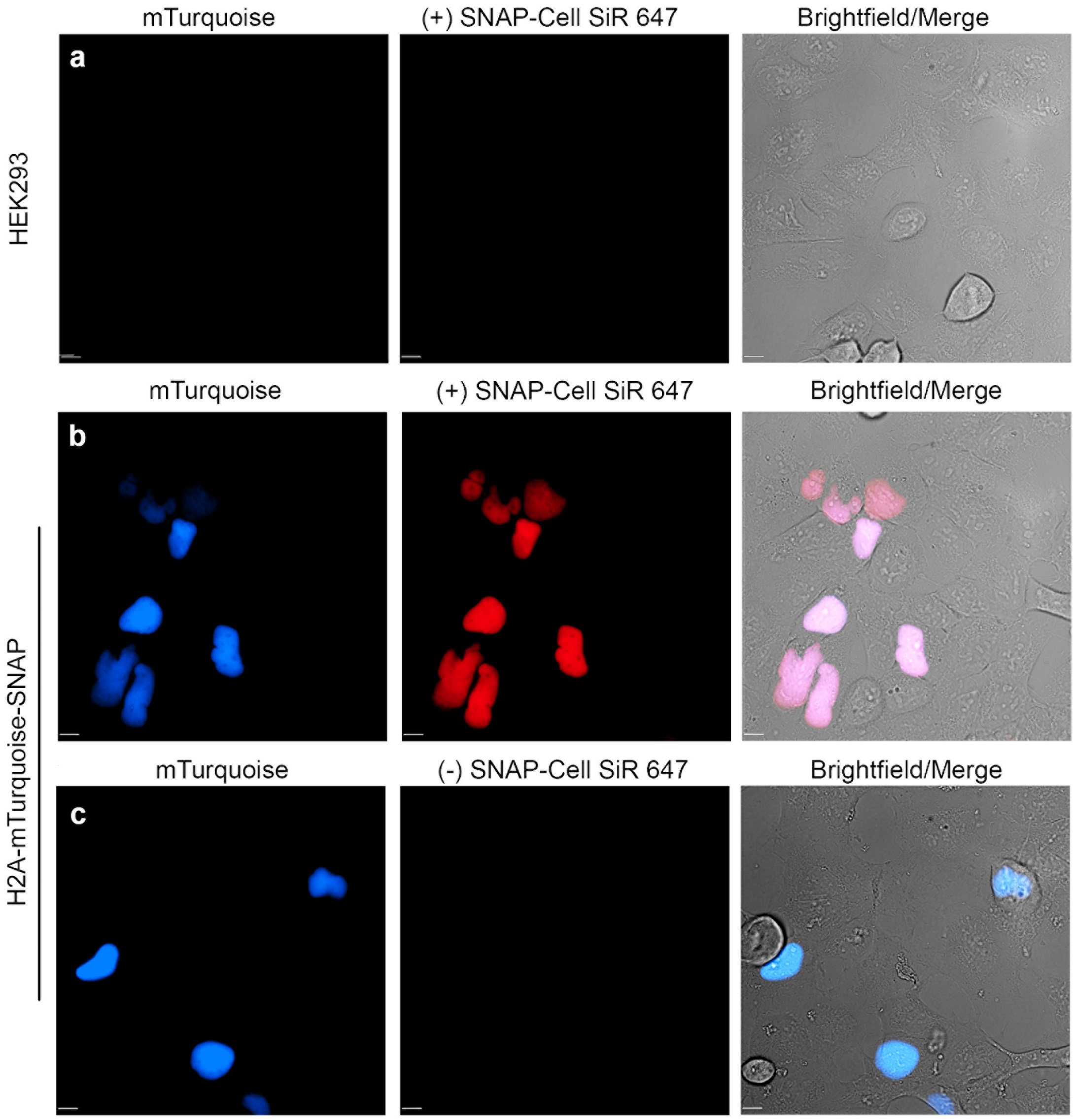

Supplementary Figure 8 – Fluorescence microscopy images of (**a**) HEK or (**b**) HEK cells transiently expressing H2A-mTurquoise-SNAP, with or (**c**) without treatment SNAP-Cell SiR 647. Brightness and contrast normalized across images, scale bar = 10 µm.

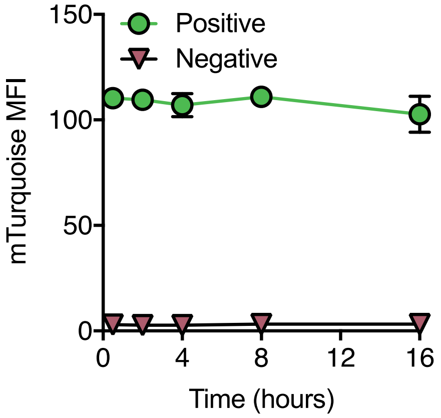

Supplementary Figure 9 – Flow cytometric analysis of HEK transiently expressing H2A-mTurquoise-SNAP over 16 hours in cells gated for positive or negative expression of mTurquoise. The mean fluorescence intensity is plotted with error bars representing the standard deviation of two experiments in duplicate (n = 4).

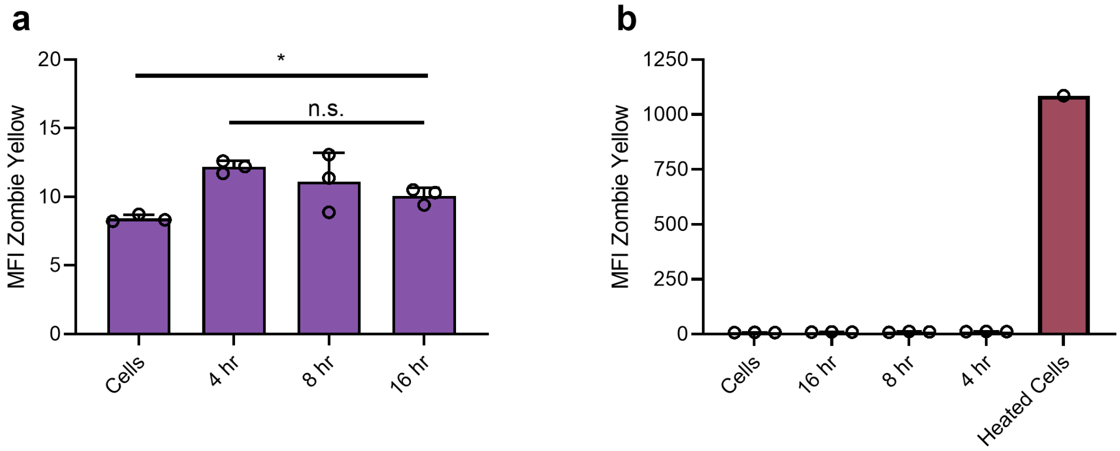

Supplementary Figure 10 – Cell viability of HEK cells by flow cytometry with Zombie Yellow. (**a**) Cells with and without Lipofectamine 3000 treatment at 4, 8 and 16 hours. (**b**) Cells in (**a**) scaled to compare to cells undergoing apoptosis induced by heat treatment at 65 °C for 5 minutes. The average mean fluorescence intensity of cells without the dye was subtracted from each sample with dye and is plotted with error bars representing the standard deviation of one experiment in triplicate (n = 3) (n.s. = non-significant, *P < 0.05, one-way ANOVA).

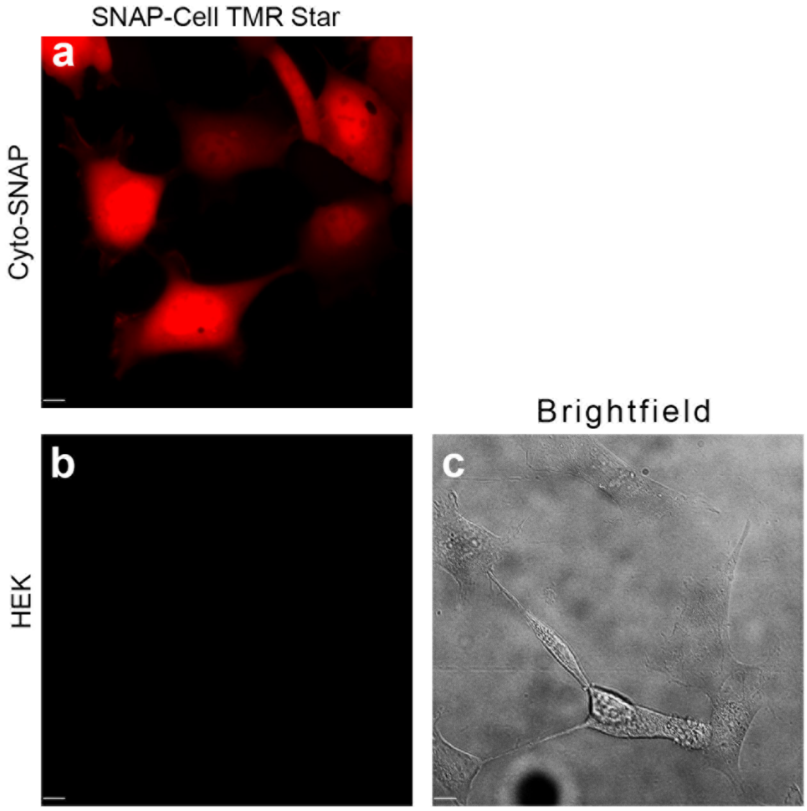

Supplementary Figure 11 – Fluorescence microscopy images of HEK293 transfected with SNAP-Cell TMR-Star for 30 minutes at 37°C with (**a**) Cyto-SNAP or (**b**) without transfection. (**c**) Brightfield image of cells in (**b**). Scale bar = 10 µm.

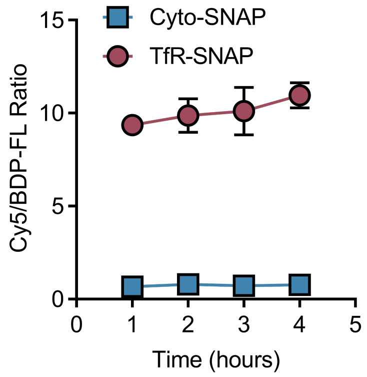

Supplementary Figure 12 – $SNAP_{Switch}$ conjugated to the anti-mouse transferrin antibody anti-mTfR is activated by SNAP-tag fused to the transferrin receptor (hTfR-SNAP) but not enzyme expressed in the cytosol (Cyto-SNAP) over 4 hours in 3T3 cells. The mean ratio is plotted with error bars representing the standard deviation of two experiments in triplicate (n = 6).

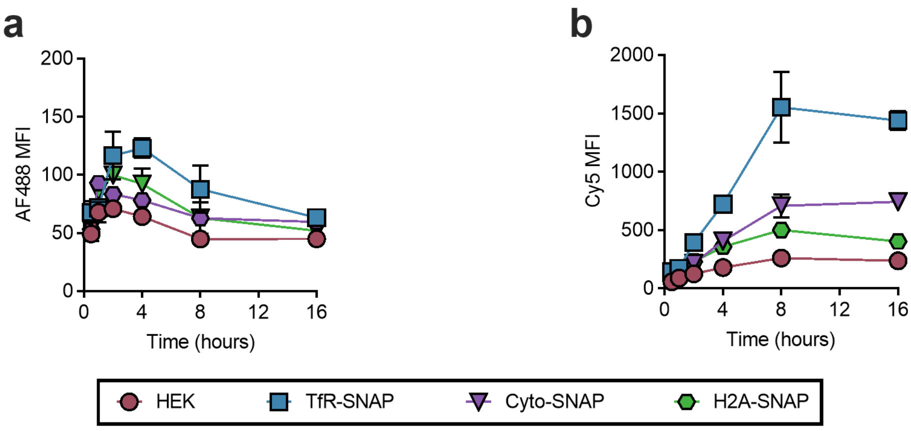

Supplementary Figure 13 – HEK cells stably expressing TfR-SNAP, Cyto-SNAP or H2A-SNAP and transfected with Lipofectamine 3000 complexed oligonucleotides labelled with both AF488 and $SNAP_{Switch}$. (**a**) The association of complexes with cells over time by flow cytometry, measured by the AF488 fluorescence intensity. (**b**) $SNAP_{Switch}$ signal over time. The mean fluorescence intensity or ratio is plotted with error bars representing the standard deviation, in triplicate (n = 3).

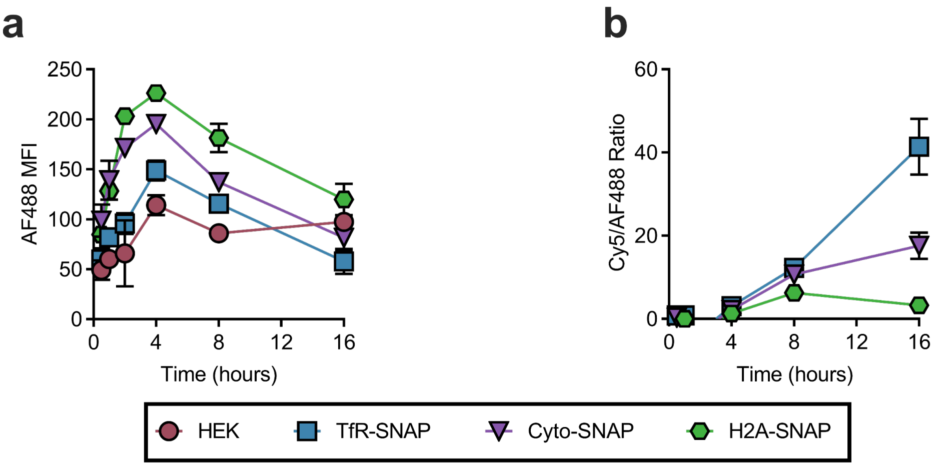

Supplementary Figure 14 – Repeat experiment of **Figure 3g** in the main text and **SI Figure 12**. HEK cells stably expressing TfR-SNAP, Cyto-SNAP or H2A-SNAP and transfected with Lipofectamine 3000 complexed oligonucleotides labelled with both AF488 and $SNAP_{Switch}$. (**a**) The association of complexes with cells over time by flow cytometry, measured by the AF488 fluorescence intensity. (**b**) The ratio of $SNAP_{Switch}$ to AF488 signal at each time point one experiment in triplicate (n = 3) with the average ratio in HEK cells subtracted from each data point as background.

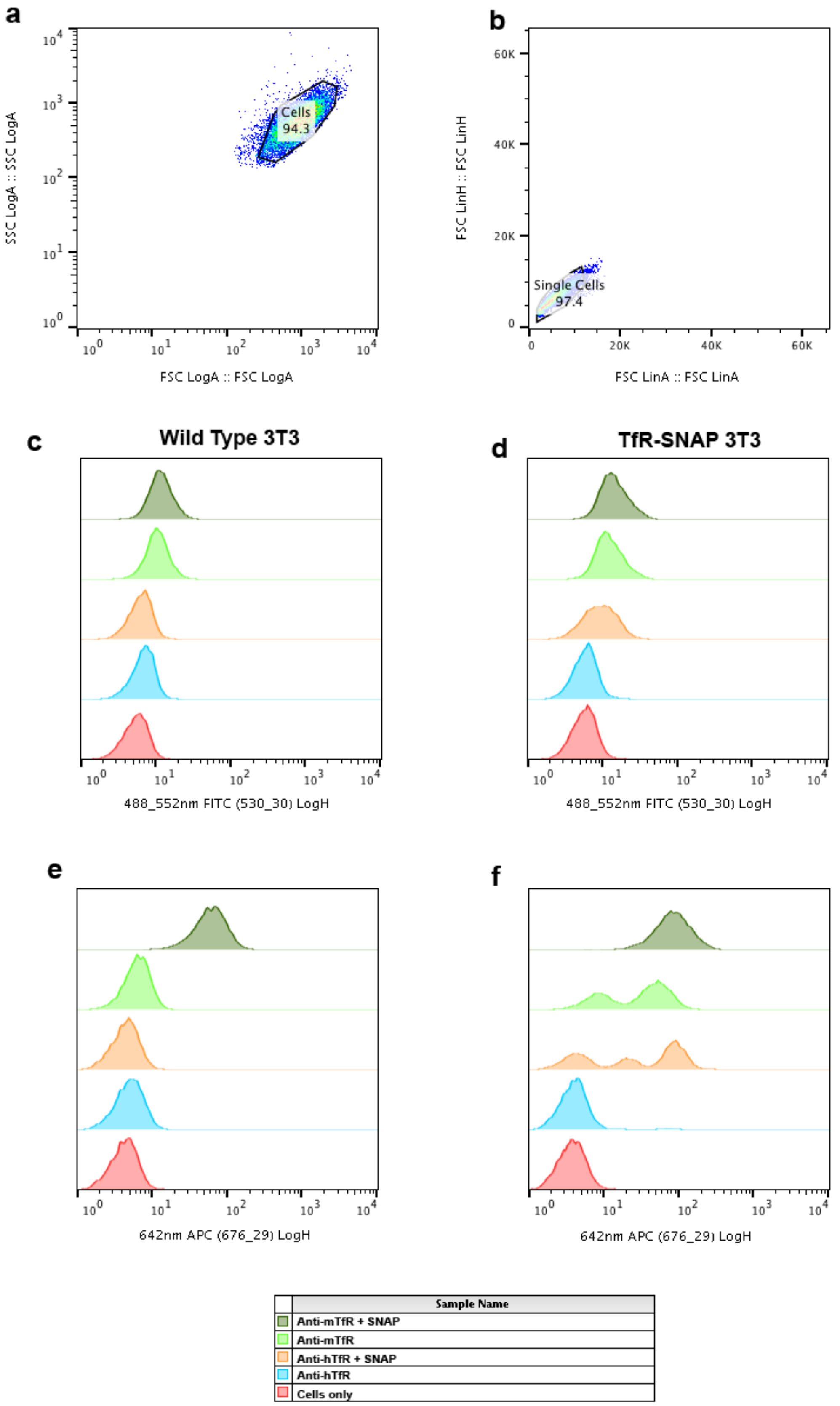

Supplementary Figure 15 – Example (data from Figure 4, main text) of the gating strategy for flow cytometry performed on 3T3 cells (Figure 3, 4 and SI Figure 7, 12). Unstained wild type or TfR-SNAP cells were gated using a (**a**) forward versus side scatter log area plot to remove debris, followed by a (**b**) forward linear height by forward linear area to remove doublets. Representative histograms of the Alexa Fluor 488 and $\text{SNAP}_{\text{Switch}}$ signal from anti-mTfR and anti-hTfR with and without SNAP-tag pre-treatment in (**c,e**) wild type 3T3 and (**d,f**) TfR-SNAP 3T3 cells.

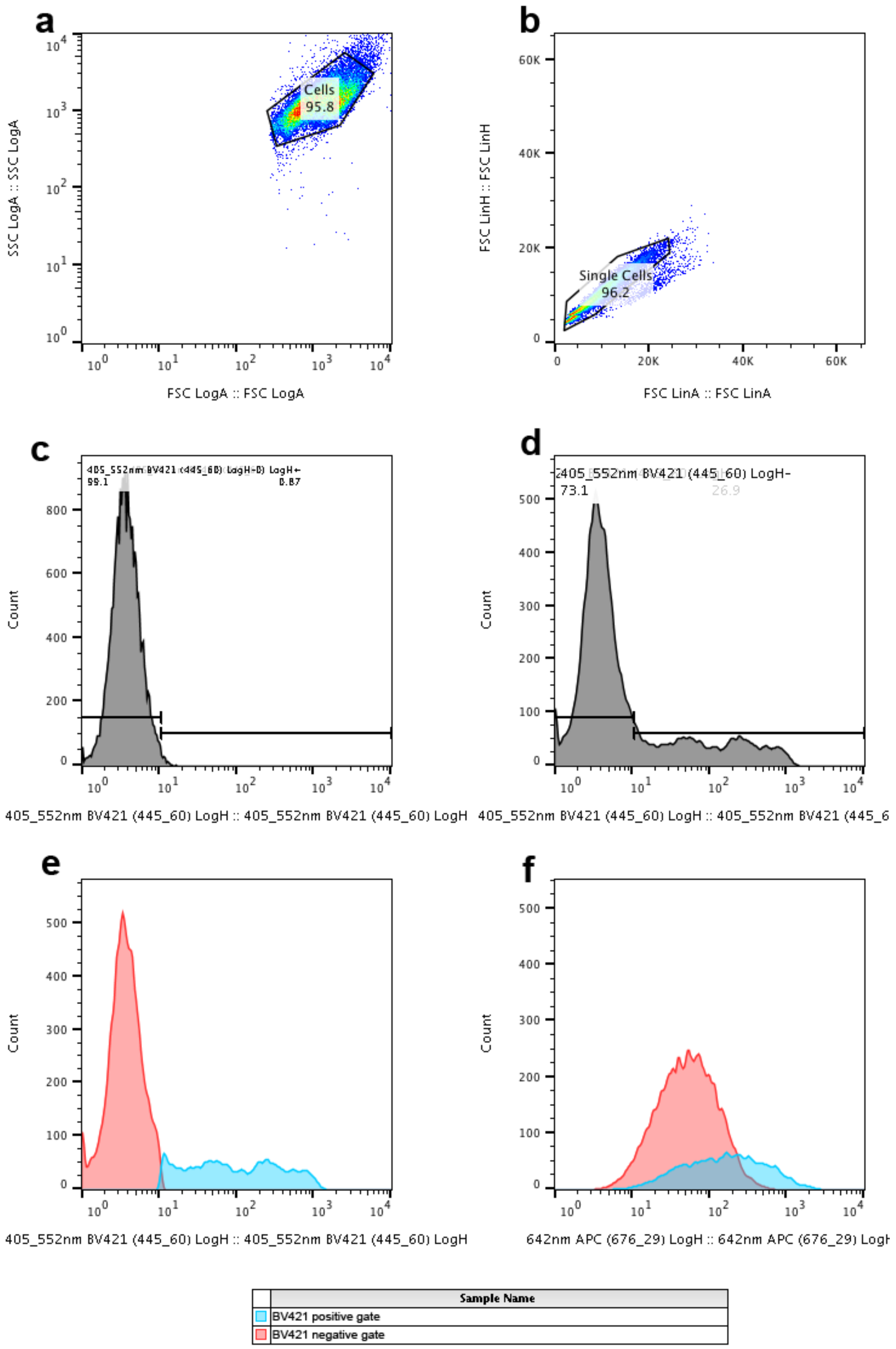

Supplementary Figure 16 – Example (data from Figure 5, main text) of the gating strategy for flow cytometry performed on HEK cells (Figure 5, SI Figure 9, 10, 13, 14). HEK cells transfected with an empty plasmid (**a**) forward versus side scatter log area plot to remove debris, followed by a (**b**) forward linear height by forward linear area to remove doublets. Within this gate, (**c**) two gates were created for mTurquoise negative and mTurquoise positive fluorescence and this was applied to (**d**) H2A-mTurq-SNAP samples. Representative histograms of the (**e**) mTurquoise signal in the positive and negative gates applied to cells transfected with H2A-mTurq-SNAP and (**f**) the $SNAP_{Switch}$ signal in the positive and negative gates for mTurquoise in cells transfected with H2A-mTurq-SNAP followed by a 16 hour incubation with the AF488/$SNAP_{Switch}$ lipoplexes.

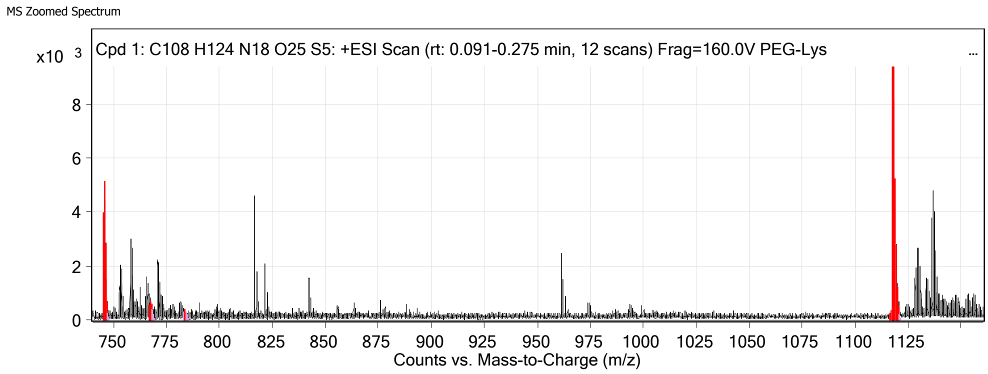

Supplementary Figure 17 – HRMS ESI$^+$ spectra of purified $SNAP_{Switch}$ with found ions highlighted in red.

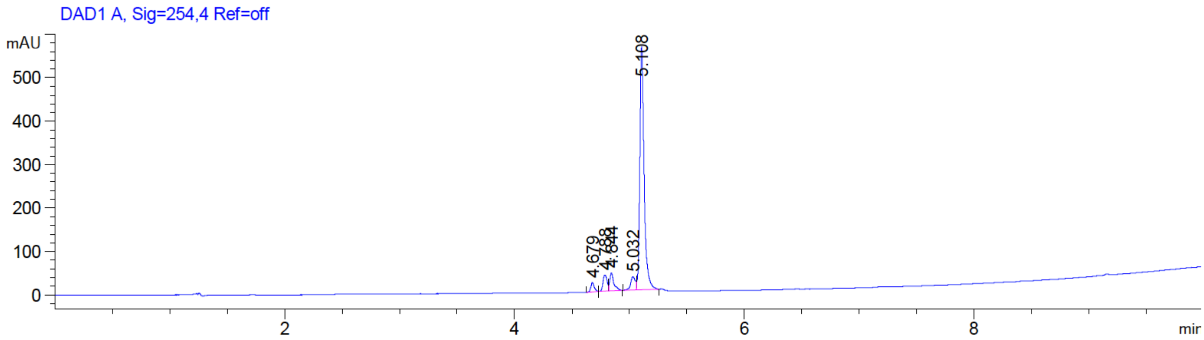

Supplementary Figure 18 – Analytical HPLC trace of purified $SNAP_{Switch}$. UV absorbance by diode array detector at 254 nm.

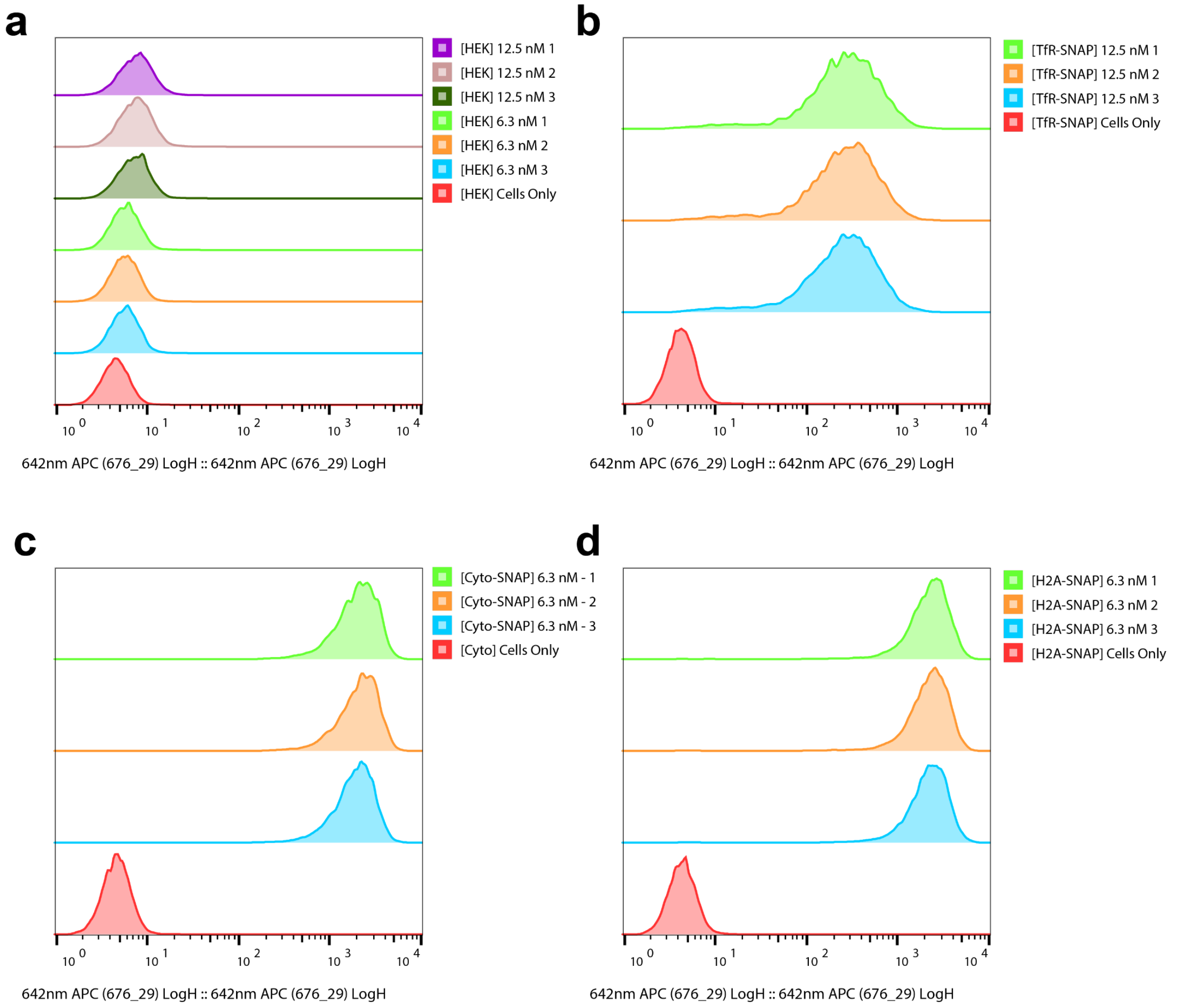

Supplementary Figure 19 – Histograms showing homogenous SNAP-tag expression in cells stably expressing the SNAP-tag. (**a**) HEK, (**b**) TfR-SNAP, (**c**) Cyto-SNAP or (**d**) H2A-SNAP cells were treated with 6.25 nM (H2A- and Cyto-SNAP) or 12.5 nM (TfR-SNAP) SNAP-Cell 647-SiR and analysed by flow cytometry.

a

b

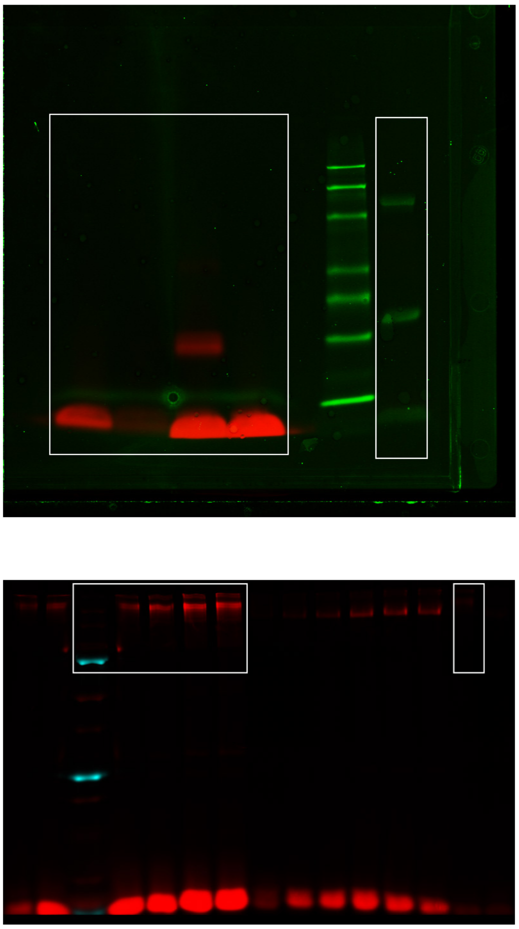

Supplementary Figure 20 – Uncropped original in-gel fluorescence scans of (**a**) Figure 2a and (**b**) SI Figure 7.
