# Supplementary material for: A molecular sensor to quantify the localization of proteins, DNA and nanoparticles in cells
Source: Nat Commun. 2020 Sep 8;11:4482. doi: 10.1038/s41467-020-18082-8 (PMC7479595; doi:10.1038/s41467-020-18082-8)
Supplement: Supplementary file 3 — Reporting Summary [file 41467_2020_18082_MOESM3_ESM.pdf]

nature research

Corresponding author(s): Angus Johnston

Last updated by author(s): Jul 23, 2020

Reporting Summary

Nature Research wishes to improve the reproducibility of the work that we publish. This form provides structure for consistency and transparency in reporting. For further information on Nature Research policies, see our Editorial Policies and the Editorial Policy Checklist.

Statistics

For all statistical analyses, confirm that the following items are present in the figure legend, table legend, main text, or Methods section.

n/a	Confirmed	
☐	☒	The exact sample size (*n*) for each experimental group/condition, given as a discrete number and unit of measurement
☐	☒	A statement on whether measurements were taken from distinct samples or whether the same sample was measured repeatedly
☐	☒	The statistical test(s) used AND whether they are one- or two-sided<br>*Only common tests should be described solely by name; describe more complex techniques in the Methods section.*
☒	☐	A description of all covariates tested
☐	☒	A description of any assumptions or corrections, such as tests of normality and adjustment for multiple comparisons
☐	☒	A full description of the statistical parameters including central tendency (e.g. means) or other basic estimates (e.g. regression coefficient) AND variation (e.g. standard deviation) or associated estimates of uncertainty (e.g. confidence intervals)
☐	☒	For null hypothesis testing, the test statistic (e.g. *F*, *t*, *r*) with confidence intervals, effect sizes, degrees of freedom and *P* value noted<br>*Give P values as exact values whenever suitable.*
☒	☐	For Bayesian analysis, information on the choice of priors and Markov chain Monte Carlo settings
☒	☐	For hierarchical and complex designs, identification of the appropriate level for tests and full reporting of outcomes
☒	☐	Estimates of effect sizes (e.g. Cohen's *d*, Pearson's *r*), indicating how they were calculated

Our web collection on statistics for biologists contains articles on many of the points above.

Software and code

Policy information about availability of computer code

Data collection	Data from flow cytometry was acquried using CellCapTure Analysis Software (version 4.0 RC4, Stratedigm, California, USA). Images from fluorescence microscopy were obtained with SlideBook 6 (Intelligent Imaging Innovations, Denver, USA). HRMS acquisition and analysis were performed with Masslynx (version 4.1, Waters Corp, Massachusetts, USA).
Data analysis	Data analysis for flow cytometry was performed using FlowJo (version 8, Tree Star, Oregon, USA). Images from microscopy were processed with SlideBook 6 (version 6.0.7 (25602), Intelligent Imaging Innovations, Denver, USA) and ImageJ (Version 1.52g, Wayne Rasband, National Institute of Health, USA). The CUDA deconvolution ImageJ plugin was also used (Bruce, M. a & Butte, M. J. Opt. Express 21, 4766–4773 (2013)). Graphs were plotted and statistics calculated using GraphPad Prism 8 (Version 8.3.0 (538), GraphPad Software Inc., USA).

For manuscripts utilizing custom algorithms or software that are central to the research but not yet described in published literature, software must be made available to editors and reviewers. We strongly encourage code deposition in a community repository (e.g. GitHub). See the Nature Research guidelines for submitting code & software for further information.

Data

Policy information about availability of data

All manuscripts must include a data availability statement. This statement should provide the following information, where applicable:

- Accession codes, unique identifiers, or web links for publicly available datasets
- A list of figures that have associated raw data
- A description of any restrictions on data availability

The source data underlying all the figures of this study are available as Source Data files. The raw data is available from the corresponding author upon request.

Field-specific reporting

Please select the one below that is the best fit for your research. If you are not sure, read the appropriate sections before making your selection.

☒ Life sciences ☐ Behavioural & social sciences ☐ Ecological, evolutionary & environmental sciences

For a reference copy of the document with all sections, see nature.com/documents/nr-reporting-summary-flat.pdf

Life sciences study design

All studies must disclose on these points even when the disclosure is negative.

Sample size	No statistics were used to determine sample size. Replicates were performed to ensure sample sizes were a true representation of the population.
Data exclusions	No data was excluded from this study.
Replication	Experiments were performed independently in at least duplicate and all attempts at replication were successful.
Randomization	For time course experiments, the order in which the Lipofectamine complexes or antibodies were added to the different cell lines was random to control for slight differences in incubation time.
Blinding	Investigators were not blinded because the analysis of the fluorescence intensity across cell types was calculated by FlowJo leaving no room for subjectivity.

Reporting for specific materials, systems and methods

We require information from authors about some types of materials, experimental systems and methods used in many studies. Here, indicate whether each material, system or method listed is relevant to your study. If you are not sure if a list item applies to your research, read the appropriate section before selecting a response.

Materials & experimental systems

n/a	Involved in the study	
☐	☒	Antibodies
☐	☒	Eukaryotic cell lines
☒	☐	Palaeontology and archaeology
☒	☐	Animals and other organisms
☒	☐	Human research participants
☒	☐	Clinical data
☒	☐	Dual use research of concern

Methods

n/a	Involved in the study	
☒	☐	ChIP-seq
☐	☒	Flow cytometry
☒	☐	MRI-based neuroimaging

Antibodies

Antibodies used	Monoclonal IgG1 anti-transferrin receptor (human, clone OKT9) and monoclonal IgG2a anti-transferrin receptor (mouse, clone were purchased from Antibody Services at the Walter and Eliza Hall Institute Biotechnology Centre, Melbourne, Australia.<br><br>Monoclonal IgG1 against mouse CD44 (5035-41.1D) was purchased from Novus Biologicals
Validation	OKT9 and TIB-219 have been validated in Sutherland, R. et al. Proc. Natl. Acad. Sci. U. S. A. (1981) and Lesley, J. Cell. Immunol. 83, 14–25 (1984), respectively.<br><br>The manufacturer references Chaudhary, N. et al. PLoS Biol. 2014, 12 (4), 1–20 for binding of 5035-41.1D to mouse CD44.

Eukaryotic cell lines

Policy information about cell lines

Cell line source(s)	NIH/3T3 were obtained from the ATCC (CRL-1658) and HEK293A (R70507) were obtained from ThermoFisher Scientific.
Authentication	None of the cell lines used were authenticated.
Mycoplasma contamination	All cell lines used were tested for mycoplasma contamination and were negative.

Commonly misidentified lines (See ICLAC register)	No commonly misidentified lines were used.

Flow Cytometry

Plots

Confirm that:

☒ The axis labels state the marker and fluorochrome used (e.g. CD4-FITC).

☒ The axis scales are clearly visible. Include numbers along axes only for bottom left plot of group (a 'group' is an analysis of identical markers).

☒ All plots are contour plots with outliers or pseudocolor plots.

☒ A numerical value for number of cells or percentage (with statistics) is provided.

Methodology

Sample preparation	Cell lines were seeded at densities and in plates indicated in the paper 1 day prior to the experiment. After incubation with the material of interest, they were washed in phosphate buffered saline (PBS) and detached with TrypLE Express Enzyme for 5 minutes at room temperature. 1% bovine serum albumin was added to each well and the cells were transferred to a 96-well plate. The cells were pelleted by spinning at 350 g for 5 minutes and resuspended in PBS.
Instrument	Stratedigm S1000EXI
Software	CellCapTure Analysis Software (version 4.0 RC4, Stratedigm, California, USA)
Cell population abundance	All cell lines were homogeneous, containing only one population.
Gating strategy	Cells were gated using the forward versus side scatter log area plot.

☒ Tick this box to confirm that a figure exemplifying the gating strategy is provided in the Supplementary Information.
